# Association between sedentary behavior and dynapenic abdominal obesity among older adults from low- and middle-income countries

**DOI:** 10.1007/s40520-024-02763-1

**Published:** 2024-05-11

**Authors:** Lee Smith, Guillermo F. López Sánchez, Masoud Rahmati, Mark A. Tully, Damiano Pizzol, Nicola Veronese, Pinar Soysal, Karel Kostev, Dong Keon Yon, Laurie Butler, Jae Il Shin, Ai Koyanagi

**Affiliations:** 1 Centre for Health Performance and Wellbeing, Anglia Ruskin University, Cambridge, UK; 2 Division of Preventive Medicine and Public Health, Department of Public Health Sciences, School of Medicine, University of Murcia, Murcia, Spain; 3 CEReSS-Health Service Research and Quality of Life Center, Aix-Marseille University, Marseille, France; 4 Department of Physical Education and Sport Sciences, Faculty of Literature and Human Sciences, Lorestan University, Khoramabad, Iran; 5 Department of Physical Education and Sport Sciences, Faculty of Literature and Humanities, Vali-E-Asr University of Rafsanjan, Rafsanjan, Iran; 6 School of Medicine, Ulster University, Londonderry, Northern Ireland, UK; 7 Italian Agency for Development Cooperation, Khartoum, Sudan; 8 Geriatric Unit, Department of Internal Medicine and Geriatrics, University of Palermo, Palermo, Italy; 9 Department of Geriatric Medicine, Faculty of Medicine, Bezmialem Vakif University, Istanbul, Turkey; 10 University Clinic of Marburg, Marburg, Germany; 11 Center for Digital Health, Medical Science Research Institute, Kyung Hee University Medical Center, Kyung Hee University College of Medicine, Seoul, Republic of Korea; 12 Department of Pediatrics, Kyung Hee University College of Medicine, Seoul, Republic of Korea; 13 Department of Pediatrics, Yonsei University College of Medicine, Seoul, Republic of Korea; 14 Severance Underwood Meta-Research Center, Institute of Convergence Science, Yonsei University, Seoul, Republic of Korea; 15 Research and Development Unit, Parc Sanitari Sant Joan de Déu, Dr. Antoni Pujadas, Sant Boi de Llobregat, Barcelona, Spain

**Keywords:** Dynapenic abdominal obesity, Sitting time, Older adults, Low- and middle-income countries, Epidemiology

## Abstract

**Background:**

Sedentary behavior, or time spent sitting, may increase risk for dynapenic abdominal obesity (DAO), but there are currently no studies on this topic.

**Aims:**

Therefore, we investigated the association between sedentary behaviour and DAO in a nationally representative sample of older adults from six low- and middle-income countries.

**Methods:**

Cross-sectional data from the Study on Global AGEing and Adult Health were analysed. Dynapenia was defined as handgrip strength < 26 kg for men and < 16 kg for women. Abdominal obesity was defined as waist circumference of > 88 cm (> 80 cm for Asian countries) for women and > 102 cm (> 90 cm) for men. DAO was defined as having both dynapenia and abdominal obesity. Self-reported sedentary behavior was categorized as ≥ 8 h/day (high sedentary behaviour) or < 8 h/day. Multivariable multinomial logistic regression was conducted.

**Results:**

Data on 20,198 adults aged ≥ 60 years were analyzed [mean (SD) age 69.3 (13.1) years; 54.1% females]. In the overall sample, ≥ 8 h of sedentary behavior per day (vs. <8 h) was significantly associated with 1.52 (95%CI = 1.11–2.07) times higher odds for DAO (vs. no dynapenia and no abdominal obesity), and this was particularly pronounced among males (OR = 2.27; 95%CI = 1.42–3.62). Highly sedentary behavior was not significantly associated with dynapenia alone or abdominal obesity alone.

**Discussion:**

High sedentary behaviour may increase risk for DAO among older adults.

**Conclusions:**

Interventions to reduce sedentary behaviour may also lead to reduction of DAO and its adverse health outcomes, especially among males, pending future longitudinal research.

**Supplementary Information:**

The online version contains supplementary material available at 10.1007/s40520-024-02763-1.

## Introduction

Dynapenic abdominal obesity (DAO) is defined as the co-existence of abdominal obesity and low muscle strength [1], and its prevalence has been reported to be high in the general population, particularly among older adults [2]. DAO is an emerging important risk concept as recent studies have shown that this condition is associated with a high risk for a myriad of adverse health outcomes including falls [3, 4], cognitive impairment [5], worsening disability, and premature mortality [6]. Given this, it is particularly important to identify risk factors of DAO among the older population to inform targeted interventions in the prevention of DAO and its adverse health outcomes.

While the specific risk factors for DAO are largely unknown, one potentially important risk factor may be sedentary behavior. Sedentary behavior refers to any waking behavior with an energy expenditure ≤ 1.5 metabolic equivalent task units (METs), while in a reclining, sitting, or lying posture [7]. Sedentary behavior could potentially increase risk for DAO as excessive sedentary time has been associated with higher risk for its individual components (i.e., central obesity and weak muscle strength) [8–10]. Specifically, reduction in energy expenditure due to excessive sedentary behavior can lead to central obesity, while sedentary behavior can potentially cause low muscle strength via absence of muscle contractile activity, and other physiological consequences on muscle metabolism [11].

While no literature exists on the relationship between sedentary behavior and DAO, a small number of studies exist on the association between sedentary behavior and sarcopenic obesity. Sarcopenic obesity is defined as low skeletal muscle mass coupled with high levels of adiposity [12], and is a similar but distinctly different concept from DAO, as low skeletal muscle mass does not always correspond with muscle strength [13]. Specifically, one cross-sectional study which included 1286 British men, aged 70 to 92 years, found that sedentary time was associated with an increased risk of sarcopenic obesity independent of levels of moderate-to-vigorous physical activity [14]. In another cross-sectional study including black South African women between the ages of 60–85 years (*n* = 122) from a low-income community, it was observed that those with sarcopenic obesity presented with a descriptive profile of higher sedentary time than women without sarcopenic obesity [15]. Of note, this latter study used body mass index (BMI) to define obesity and not indicators of central obesity. Research suggests that BMI does not differentiate between lean body mass and fat mass; therefore, when using BMI as a measure, inaccurate assessment of adiposity is possible [16]. Moreover, existing evidence suggests that central obesity and abdominal deposition of fat are more strongly associated with a plethora of chronic diseases compared to BMI [17].

Given this background, the aim of the present study was to investigate the association between sedentary behavior and DAO in a sample of 20,198 adults aged ≥ 60 years from six low- and middle-income countries (LMICs). DAO may be particularly problematic in LMICs as the speed of ageing is surpassing that of high-income countries in this setting [18], while obesity and dynapenia are likely to be increasing due to changes in lifestyles (e.g., change in dietary habits, greater sedentary behavior).

## Methods

### The survey

We analyzed data from the Study on Global Ageing and Adult Health (SAGE), which is a publicly available dataset. The principal aim of the survey was to collect information on adult health and wellbeing (mainly older adults) to address the gap in scientific knowledge and reliable data on health and ageing in LMICs. This survey was undertaken in China, Ghana, India, Mexico, Russia, and South Africa between 2007 and 2010. These countries represent diverse geographical locations as well as economic development, and demographic and health transition. Of note, two of the most populous countries in the world (i.e., China and India) were included in the survey. Based on the World Bank classification at the time of the survey, Ghana and India were a low-income country and a lower middle-income country, respectively, while Mexico, Russia, and South Africa were all upper middle-income countries. China was a lower middle-income country when the survey began but became an upper middle-income country in 2010. Details of the survey methodology are provided elsewhere [19]. Briefly, to obtain nationally representative samples, a multistage clustered sampling design method was utilized. With the exception of Mexico, which used a similar but slightly different sampling method, households were classified into two mutually exclusive groups: (a) everyone aged ≥ 50 years were selected from households named “50 + households”, and were invited to complete the individual interview; and (b) a single individual aged 18–49 years was selected from the “18–49 household” for the individual interview. Thus, the sample consisted of adults aged ≥ 18 years including an oversampling of people aged ≥ 50 years. In order to obtain the final sampling units, household enumerations were conducted. Trained interviewers performed face-to-face interviews using a standard questionnaire. Standard translation procedures were undertaken to ensure comparability of the questionnaire between countries. Computer-assisted personal interviews (CAPI) were utilized in half of the interviews in China, while the other half was completed using paper and pencil. Mexico only utilized CAPI, while the other four countries only used paper and pencil format. On average, the duration of the interview was 2.5 h. The survey response rates were: China 93%; Ghana 81%; India 68%; Mexico 53%; Russia 83%; and South Africa 75%. In Mexico, response rates were lowest, possibly due to the short time available for field visits, which did not allow for enough re-visits if the participant was not at home. Sampling weights were created to adjust for the population structure based on information from the United Nations Statistical Division. Ethical approval was obtained from the WHO Ethical Review Committee as well as local ethics research review boards. Written informed consent was obtained from all participants.

### Sedentary behavior

To assess sedentary behavior, participants were asked to state how much time they spent usually (expressed in minutes per day) sitting or reclining in total including at work, getting to and from places, at home, or with friends (sitting with friends, sitting at a desk, travelling in bus, car, train, reading, watching television or playing cards). Time spent sleeping was excluded. This single question is derived from the Global Physical Activity Questionnaire (GPAQ) [20]. The variable on sedentary behavior was used as continuous variable, and also as a dichotomous variable (< 8 or ≥ 8 h per day (i.e., highly sedentary)) in the analysis. The eight hours cut-off was selected as previous research showed that being sedentary for ≥ 8 h/day is associated with a higher risk for premature mortality [21].

### Dynapenia, abdominal obesity, and dynapenic abdominal obesity

Handgrip strength was measured with a Smedley Hand Dynamometer (Scandidact Aps, Denmark). The definition of dynapenia was < 26 kg for men and < 16 kg for women [22], using the mean value of the two handgrip measurements of the dominant hand. Waist circumference was measured at the midpoint between the lower margin of the least palpable rib and the top of the iliac crest while keeping the measuring tape parallel to the floor. Abdominal obesity was defined as a waist circumference of > 88 cm for women and > 102 cm for men for non-Asian countries (i.e., Ghana, Mexico, Russia, South Africa) [23]. For Asian countries (i.e., China, India), abdominal obesity referred to a waist circumference of > 80 cm for women and > 90 cm for men [24]. Participants were classified into four groups according to dynapenia and abdominal obesity status: (a) No dynapenia and no abdominal obesity; (b) dynapenia alone; (c) abdominal obesity alone; and (d) dynapenia and abdominal obesity (i.e., DAO).

### Control variables

The selection of the control variables was based on past literature [25, 26] and included age, sex, highest level of education achieved (≤ primary, secondary, tertiary), country-wise wealth quintiles based on income, marital status (currently married/cohabiting or else), setting (urban or rural), smoking (never, current, past), alcohol consumption, fruit/vegetable consumption, physical activity, number of chronic physical conditions, and disability. Consumers of at least four (females) or five drinks (males) of any alcoholic beverage per day on one day or more in the past week were considered ‘heavy’ drinkers. Those who had ever consumed alcohol but were not heavy drinkers were considered ‘non-heavy’ drinkers [27]. Participants were asked the two following questions: “How many servings of fruit do you eat on a typical day?” and “How many servings of vegetables do you eat on a typical day?”Those who consumed ≥ 2 servings of fruits and ≥ 3 servings of vegetables were considered to have adequate fruit/vegetable consumption [28]. Levels of physical activity were assessed with the Global Physical Activity Questionnaire and were categorized as low, moderate, and high based on conventional cut-offs [20]. Information on 11 chronic physical diseases (angina, arthritis, asthma, chronic back pain, chronic lung disease, diabetes, edentulism, hearing problem, hypertension, stroke, visual impairment) were collected. The details on the diagnosis of these conditions are shown in Table S1 (Appendix). The number of chronic conditions were summed per participant and categorized as 0, 1, and ≥ 2. Standard basic ADL questions were used to assess the level of disability [29–31]. The six questions on ADL had the introductory phrase “overall in the last 30 days, how much difficulty did you have” followed by: in washing your whole body?; in getting dressed?; with moving around inside your home?; with eating (including cutting up your food)?; with getting up from lying down?; with getting to and using the toilet? Answer options included none, mild, moderate, severe, extreme/cannot do. Disability was a dichotomous variable where those who answered severe or extreme/cannot do to any of the six questions were considered to have ADL disability [32].

### Statistical analysis

The statistical analysis was performed with Stata 14.1 (Stata Corp LP, College station, Texas). Multivariable multinomial logistic regression analysis was conducted to assess the association between sedentary behavior (exposure) and the four-category variable on dynapenia, abdominal obesity, or both (outcome), with no dynapenia and no abdominal obesity being the base category for the outcome. Sedentary behavior was used in the analysis as a dichotomous variable (i.e., < 8 and ≥ 8 h/day) or as a continuous variable (hours/day). Analyses using the overall sample and sex-stratified samples were conducted. The models were adjusted for age, sex, education, wealth, marital status, setting, smoking, alcohol consumption, fruit/vegetable consumption, physical activity, number of chronic physical conditions, disability, and country, except for the sex-wise analysis which was not adjusted for sex. Adjustment for country was done by including dummy variables for each country in the model as in previous SAGE publications [27, 33]. The sample weighting and the complex study design were taken into account in all analyses. Results from the regression analyses are presented as odds ratios (ORs) with 95% confidence intervals (CIs). The level of statistical significance was set at two-sided *P* < 0.05.

## Results

**Fig. 1:**
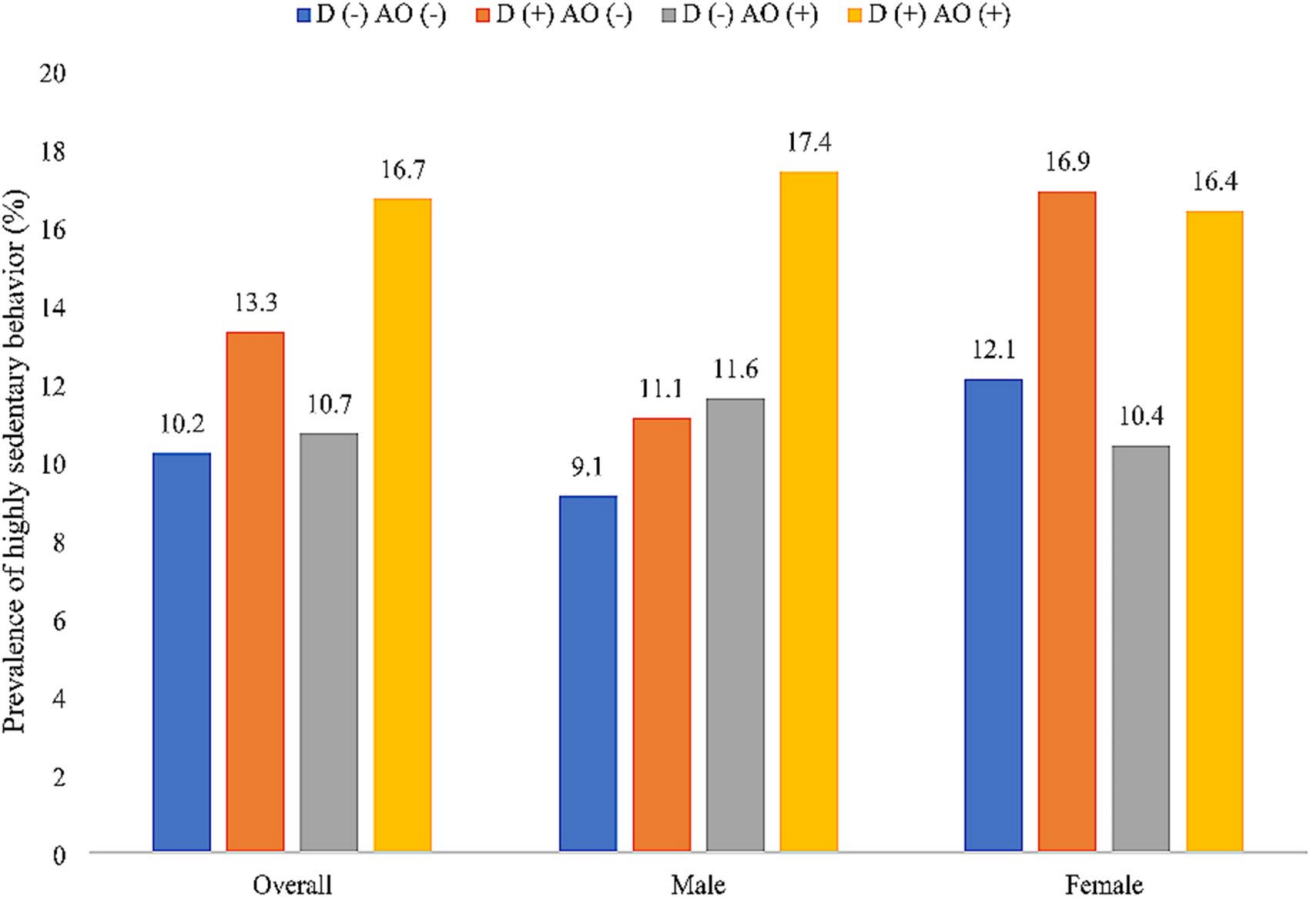
Prevalence of highly sedentary behavior (i.e., ≥ 8 h/day) by dynapenia/abdominal obesity status (overall and by sex). *Abbreviation* D Dynapenia; AO Abdominal obesity

A total of 20,198 adults aged ≥ 60 years were included in the analysis. The sample size of each country was: China *n* = 7474; Ghana *n* = 2616; India *n* = 3621; Mexico *n* = 1879; Russia *n* = 2465; South Africa *n* = 2143. The prevalence of high sedentary behavior (i.e., ≥ 8 h/day) was 13.7%, while the prevalence of dynapenia alone, abdominal obesity alone, and DAO were 25.9%, 27.0%, and 15.1%, respectively. The sample characteristics are provided in Table 1. The mean (SD) age was 69.3 (13.1) years and 54.1% were females. The prevalence of highly sedentary behavior by dynapenia/abdominal obesity status is shown in Fig. 1. Overall, the prevalence of highly sedentary behavior was particularly high among people with DAO (16.7%), but different patterns were observed between men and women. Specifically, DAO alone was associated with a particularly high prevalence of highly sedentary behavior among men but among women, this prevalence was high not only for DAO but also dynapenia alone. Multivariable multinomial logistic regression showed that in the overall sample, ≥ 8 h of sedentary behavior per day (vs. <8 h) was significantly associated with 1.52 (95%CI = 1.11–2.07) times higher odds for DAO (vs. no dynapenia and no abdominal obesity), and this was particularly pronounced among males (OR = 2.27; 95%CI = 1.42–3.62) (Table 2). Eight hours or more of sedentary behavior was not significantly associated with dynapenia alone or abdominal obesity in the overall sample and in the sex-stratified samples, while this was not significantly associated with DAO among females. When the continuous sedentary behavior variable was used (i.e., hours/day), a one-hour increase in sedentary behavior was associated with a significant 1.08 (95%CI = 1.03–1.13) times higher odds for DAO (vs. no dynapenia and no abdominal obesity) in the overall sample, and this was again more pronounced among males (OR = 1.13; 95%CI = 1.06–1.20) (Table 3). In the overall sample and the sample restricted to males, a one-hour increase in sedentary behavior was also associated with a significant 1.06–1.07 times higher odds for dynapenia alone. Sedentary behavior was not significantly associated with abdominal obesity alone (in the overall sample and sex-stratified samples), while increasing sedentary behavior was not significantly associated with dynapenia alone and DAO either among females.

**Table 1:** Sample characteristics (overall and by dynapenia/abdominal obesity status)

Characteristic		Total	D (-) AO (-)	D (+) AO (-)	D (-) AO (+)	D (+) AO (+)
Age (years)	Mean (SD)	69.3 (13.1)	67.8 (12.5)	70.4 (13.0)	67.8 (12.1)	70.5 (12.8)
Sex	Female	54.1	37.5	38.4	74.3	74.2
	Male	45.9	62.5	61.6	25.7	25.8
Education	≤Primary	63.9	62.5	78.4	61.8	68.3
	Secondary	29.8	31.2	17.9	31.6	25.2
	Tertiary	6.3	6.3	3.7	6.6	6.4
Wealth	Poorest	20.1	21.3	26.6	14.5	15.7
	Poorer	20.2	20.5	22.3	16.8	17.3
	Middle	20.5	20.6	20.4	20.9	19.4
	Richer	18.8	19.6	16.3	22.4	21.3
	Richest	20.3	18.0	14.4	25.3	26.3
Marital status	Married/cohabiting	66.8	73.2	70.6	66.1	64.1
	Else	33.2	26.8	29.4	33.9	35.9
Setting	Urban	48.6	43.7	32.1	57.7	53.5
	Rural	51.4	56.3	67.9	42.3	46.5
Smoking	Never	60.6	48.9	46.2	74.6	74.3
	Current	31.6	42.2	45.6	19.4	19.3
	Past	7.8	8.9	8.2	6.1	6.4
Alcohol consumption	Never	67.8	59.9	72.6	70.0	81.0
	Non-heavy	29.2	35.0	24.9	27.8	17.4
	Heavy	3.0	5.1	2.5	2.2	1.6
Fruit/vegetable consumption	Adequate	32.1	30.8	20.7	38.8	39.2
	Not adequate	67.9	69.2	79.3	61.2	60.8
Physical activity	High	40.0	49.6	36.8	45.2	28.7
	Moderate	24.8	24.6	24.3	26.2	29.1
	Low	35.2	25.8	38.9	28.6	42.2
No. of chronic conditions	0	16.8	23.1	18.7	12.2	11.3
	1	30.6	36.4	29.4	32.5	27.3
	≥ 2	52.6	40.5	51.9	55.4	61.4
Disability	No	89.9	94.0	87.5	93.3	88.6
	Yes	10.1	6.0	12.5	6.7	11.4

*Abbreviation* D Dynapenia; AO Abdominal obesity; SD Standard deviation

Data are % unless otherwise stated

**Table 2:** Association between highly sedentary behavior (i.e., ≥ 8 h/day) and dynapenia, abdominal obesity, or both estimated by multivariable multinomial logistic regression (overall and by sex)

Outcome	Overall	Male	Female
D (-) AO (-)	1.00	1.00	1.00
D (+) AO (-)	1.15	1.14	1.17
	[0.83,1.60]	[0.82,1.57]	[0.65,2.10]
D (-) AO (+)	0.96	1.28	0.79
	[0.74,1.25]	[0.82,2.02]	[0.56,1.10]
D (+) AO (+)	1.52*	2.27**	1.21
	[1.11,2.07]	[1.42,3.62]	[0.82,1.79]

*Abbreviation* D Dynapenia; AO Abdominal obesity

Data are odds ratio [95% confidence interval]

Models are adjusted for age, education, wealth, marital status, setting, smoking, alcohol consumption, fruit/vegetable consumption, physical activity, number of chronic physical conditions, disability, and country

* *p* < 0.01, ** *p* < 0.001

**Table 3:** Association between hours/day of sedentary behavior and dynapenia, abdominal obesity, or both estimated by multivariable multinomial logistic regression (overall and by sex)

Outcome	Overall	Male	Female
D (-) AO (-)	1.00	1.00	1.00
D (+) AO (-)	1.06*	1.07*	1.04
	[1.02,1.10]	[1.03,1.12]	[0.99,1.10]
D (-) AO (+)	1.00	1.03	0.97
	[0.96,1.03]	[0.96,1.10]	[0.93,1.02]
D (+) AO (+)	1.08*	1.13**	1.05
	[1.03,1.13]	[1.06,1.20]	[0.99,1.11]

*Abbreviation* D Dynapenia; AO Abdominal obesity

Data are odds ratio [95% confidence interval]

Models are adjusted for age, education, wealth, marital status, setting, smoking, alcohol consumption, fruit/vegetable consumption, physical activity, number of chronic physical conditions, disability, and country. Analysis using the overall sample was also adjusted for sex

Estimates are change in odds for the outcome associated with a one-hour increase in sedentary behavior per day

* *p* < 0.01, ** *p* < 0.001

## Discussion

### Main findings

In our nationally representative study including more than 20,000 adults aged ≥ 60 years from six LMICs, we found that the prevalence of highly sedentary behavior (i.e., ≥ 8 h/day) (13.7%) and DAO (15.1%) were high. Furthermore, multivariable multinomial logistic regression showed that in the overall sample, excessive sedentary behavior was significantly associated with 1.52 (95%CI = 1.11–2.07) times higher odds for DAO (vs. no dynapenia and no abdominal obesity), and this was particularly pronounced among males (OR = 2.27; 95%CI = 1.42–3.62). When the sedentary behavior variable was used as a continuous variable (hours/day), increasing hours spent in sedentary behavior was significantly associated with increased odds for DAO in the overall sample and males, but not females. To the best of the authors’ knowledge, this is the first study to investigate the association between sedentary behavior and DAO.

### Interpretation of the findings

Our novel finding that sedentary behavior is associated with an increase in odds for DAO is indeed an important finding as DAO has been shown to be related with multiple detrimental health outcomes, including, for example, cognitive impairment, falls, disability, and premature mortality [3, 5, 6].

There are several plausible mechanisms that likely explain why sedentary behavior is associated with higher odds for DAO. First, sedentary behavior is characterized by a low energy expenditure, and when intake exceeds energy expenditure, fat accumulation occurs [34], especially in the abdominal region in the case of older adults [35]. Second, sedentary behavior could potentially reduce muscular strength through lack of muscle contractile activity, and other physiological consequences on muscle metabolism (e.g. reduces muscle glucose) [11]. This is particularly problematic in older adults as there is a natural loss of muscle strength with ageing [36]. Interestingly, in the present study, highly sedentary behavior was only associated with DAO and not dynapenia alone nor abdominal obesity alone. This finding suggests that when one exhibits excessive sedentary behavior, these two conditions likely develop simultaneously and not alone. For example, obesity can increase risk for weak muscle strength as fatty infiltration of muscle can cause low muscle strength, while abdominal obesity can potentially decrease muscle strength via endocrine and inflammatory mechanisms [37].

Another important finding from the present study was that the relationship between sedentary behavior and DAO was only significant in men. While the reason for this sex difference is unclear, it could be related to difference in body fat distribution. Indeed, males are known to accumulate more fat in the abdominal cavity than females, and this fat accumulation starts at a younger age [38]. In addition, age-related loss of muscle strength has been reported to be more pronounced in males than females and also commences at a younger age in males [39]. Previous studies have shown that abdominal obesity can lead to a reduction in hormonal and neural trophic aspects in the muscles, via chronic inflammation and reduction in tolerance to glucose [40]. Thus, it could be that males are more likely to accumulate abdominal fat due to sedentary behavior than females, and that this can lead to more pronounced muscle weakness than in females.

### Implication of the study findings

Findings from the present study suggest that interventions to reduce sedentary behavior among older adults may aid in the prevention of DAO and its associated adverse health outcomes, particularly in males. One such approach may be to utilize self-regulatory strategies (e.g., setting goals, problem-solving and planning, providing normative feedback), as such strategies have been shown to lead to declines in sedentary behavior among older adults [41]. Moreover, interventions to displace sedentary behavior with physical activity and strength training may be particularly effective as physical activity and strength training have been found to reduce central adiposity and slow down the natural decline of age-related muscle atrophy and strength [42–46].

### Strengths and limitations

The analysis of large representative samples of older adults from six LMICs and the novel investigation of the sedentary behavior-DAO association are clear strengths of the present work. However, findings must be interpreted in light of the study’s limitations. First, the study was cross-sectional in nature, and therefore, the direction of the association cannot be determined. For example, it is also possible that people with DAO prefer to be sedentary due to factors such as fear of falling. Second, the variable on sedentary behavior was based on self-report, and thus, recall bias is possible. Future studies should consider using objective measures of sedentary behavior. Third, we only had limited information regarding dietary factors (i.e., fruit and vegetable consumption), and thus, we were unable to assess the influence of other dietary factors in the association between sedentary behavior and DAO, such as total caloric intake. Fourth, given that institutionalized people were not included in the survey, our study results cannot be generalized to this population that could have higher prevalence of sedentary behavior and DAO. Finally, the survey was undertaken between 2007 and 2010. The social and economic conditions of the six nations investigated have changed since this time, and thus, it is not known whether the same associations would be observed if the study was repeated currently. However, considering that the underlying mechanism of the association between sedentary behaviour and DAO is likely to be predominantly physiological, it could be speculated that the association has not changed drastically in recent years.

## Conclusion

In the present study including large representative samples of older adults from six LMICs, it was found that sedentary behavior is associated with increased odds for DAO. Interventions to reduce sedentary behavior among older adults may have the additional benefit of preventing DAO and its adverse health outcomes. However, future longitudinal or interventional studies are needed to make concrete recommendations.

### Electronic supplementary material

Below is the link to the electronic supplementary material.

Supplementary Material 1

## Data Availability

No datasets were generated or analysed during the current study.
